# *MicroRNA-335*/*ID4* dysregulation predicts clinical outcome and facilitates leukemogenesis by activating PI3K/Akt signaling pathway in acute myeloid leukemia

**DOI:** 10.18632/aging.101991

**Published:** 2019-05-30

**Authors:** Jing-dong Zhou, Xi-xi Li, Ting-juan Zhang, Zi-jun Xu, Zhi-hui Zhang, Yu Gu, Xiang-mei Wen, Wei Zhang, Run-bi Ji, Zhao-qun Deng, Jiang Lin, Jun Qian

**Affiliations:** 1 Department of Hematology, Affiliated People’s Hospital of Jiangsu University, Zhenjiang, Jiangsu, People’s Republic of China; 2 Zhenjiang Clinical Research Center of Hematology, Zhenjiang, Jiangsu, People’s Republic of China; 3 The Key Lab of Precision Diagnosis and Treatment in Hematologic Malignancies of Zhenjiang City, Zhenjiang, Jiangsu, People’s Republic of China; 4 Department of Hematology, The Second Affiliated Hospital of Soochow University, Suzhou, Jiangsu, People’s Republic of China; 5 Laboratory Center, Affiliated People’s Hospital of Jiangsu University, Zhenjiang, Jiangsu, People’s Republic of China; 6 Department of Geriatrics, The Second Affiliated Hospital of Soochow University, Suzhou, Jiangsu, People’s Republic of China; * Equal contribution

**Keywords:** *MiR-335*, *ID4*, acute myeloid leukemia, prognosis, PI3K/Akt pathway

## Abstract

*MircoRNA-335* (*miR-335*) has been reported as a significant cancer-associated microRNA, which was often epigenetically silenced and acted as a tumor suppressor gene in diverse human solid tumors. Conversely, recent studies show that *miR-335* overexpression was identified in both adult and pediatric acute myeloid leukemia (AML), suggesting that it might play an oncogenic role of *miR-335* in AML. However, the role of *miR-335* during leukemogenesis remains to be elucidated. *MiR-335*/*ID4* expression was detected by real-time quantitative PCR and/or western blot. Survival analysis was performed to explore the association between *miR-335*/*ID4* expression and the prognosis, and further validated by public databases. Gain-of-function experiments determined by cell proliferation, apoptosis, and differentiation were conducted to investigate the biological functions of *miR-335*/*ID4*. Herein, we found that *miR-335* expression, independent of its methylation, was significantly increased and negatively correlated with reduced *ID4* expression in AML. Moreover, aberrant *miR-335*/*ID4* expression independently affected chemotherapy response and leukemia-free/overall survival in patients with AML. Gain-of-function experiments in vitro showed the oncogenic role of *miR-335* by affecting cell apoptosis and proliferation in AML, and could be rescued by *ID4* restoration. Mechanistically, we identified and verified that *miR-335/ID4* contributed to leukemogenesis through activating PI3K/Akt signaling pathway. Collectively, aberrant *miR-335*/*ID4* expression was an independent prognostic biomarker in AML. *MiR-335*/*ID4* dysregulation facilitated leukemogenesis through the activation of PI3K/Akt signaling pathway.

## INTRODUCTION

Acute myeloid leukemia (AML), the most common adult leukemia, is an aggressive blood cancer with variable clinical outcome [1, 2]. The pathogenesis of AML is a complex process including diverse molecular events and signaling pathways [3]. Despite recent advances in the treatment of leukemia including targeted drugs, the overall prognosis for AML remains unsatisfactory [1, 2]. The karyotypes together with age and white blood cells of AML patients assessed at diagnosis are generally recognized as the three main prognostic factors [4, 5]. Recently, mounting evidences showed that molecular biomarkers such as mutations in *NPM1*, *CEBPA*, *FLT3*, and *C-KIT* as well as *BAALC*, *MN1*, *EVI1*, and *ERG* overexpression also provide powerful prognostic information [6]. Therefore, understanding molecular mechanism and finding effective prognostic biomarkers has been being one of the most urgent clinical needs and research hotspots.

MicroRNAs (miRNAs) are small non-coding RNAs that post-transcriptionally regulate gene expression, often targeting hundreds of different mRNAs with both temporal and spatial specificity [7, 8]. Increasing data show that many types of cancers were accompanied with the dysregulation of miRNAs, which contributing to tumorigenesis through various critical processes, including cell differentiation, apoptosis, proliferation and hematopoiesis [9, 10]. Notably, individual miRNAs may play distinct roles in cancers originating from different tissues or even from different lineages of the same cancer [11]. *MircoRNA-335* (*miR-335*) has been reported as a significant cancer-associated miRNA, which was often epigenetically downregulated and acted as a tumor suppressor gene in diverse human cancers [12]. Interestingly, two recent studies showed that overexpression of *miR-335* was identified in both adult and pediatric AML, and correlated with poor clinical outcome [13, 14]. However, the potential role of *miR-335* in AML remains to be elucidated.

In the present study, we first validated that *miR-335* expression was significantly increased and negatively correlated with decreased *ID4* expression in AML. Moreover, aberrant *miR-335*/*ID4* expression independently affected chemotherapy response and survival in patients with AML. Next, functional experiments in vitro showed the oncogenic role of *miR-335* in AML, and could be rescued by *ID4*. Lastly, we identified that *miR-335* contributed to leukemogenesis through PI3K/Akt signaling pathway.

## RESULTS

### MiR-335 overexpression negatively correlated with ID4 underexpression in AML

**Figure 1:**
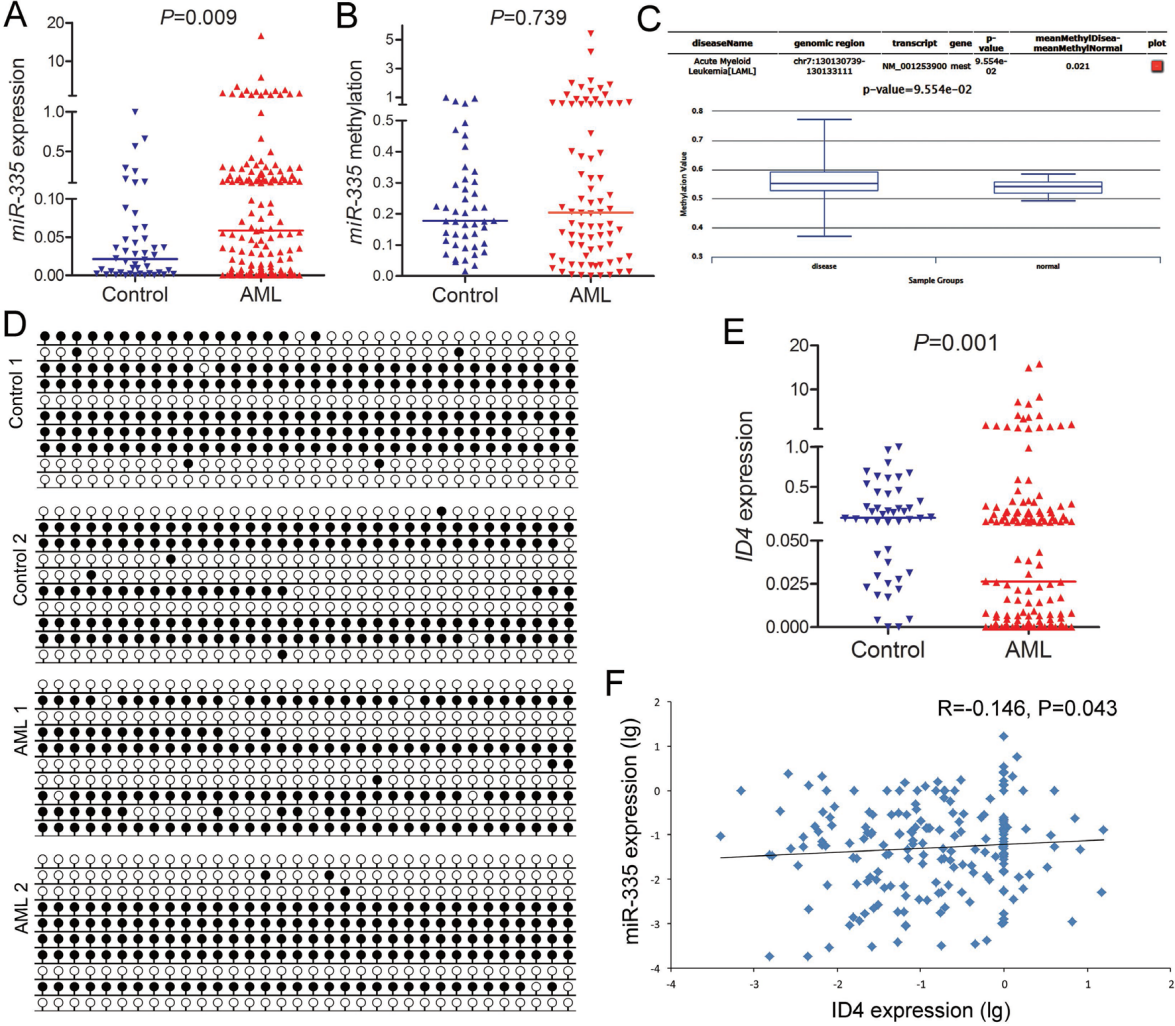
***MiR-335* overexpression negatively correlated with *ID4* underexpression in AML.** (**A**) *MiR-335* expression in controls and AML patients. *MiR-335* expression examined by RT-qPCR was significantly upregulated in AML patients. (**B**) *MiR-335* methylation level in controls and AML patients. *MiR-335* methylation examined by RQ-MSP in AML patients was similar to controls. (**C**) *MiR-335* methylation level in controls and AML patients obtained by bioinformatics analysis. *MiR-335* promoter (CpG island) methylation level was obtained through the human disease methylation database DiseaseMeth version 2.0 (http://www.bio-bigdata.com/diseasemeth/analyze.html). (**D**) *MiR-335* methylation density in controls and representative AML patients. *MiR-335* methylation density was determined by BSP. Control 1 and Control 2 indicated two controls were similar to AML 1 and AML 2 represented two AML patients. (**E**) *ID4* expression level in controls and AML patients. *ID4* expression examined by RT-qPCR was significantly down-regulated in AML patients. (**F**) Correlation between *miR-335* and *ID4* expression in AML. A negative correlation was observed between *miR-335* and *ID4* expression. The correlation analysis was conducted by Spearman test.

Mounting studies have demonstrated the tumor suppressor role of *miR-335* in solid tumors, and *miR-335* expression was epigenetically silenced by DNA methylation [15–17]. Herein, we found that *miR-335* expression was significantly upregulated in AML patients compared to healthy donors (Figure 1A). Moreover, we further analyzed the methylation pattern in the CpG island near *miR-335*. In contrast to the solid tumors, *miR-335* methylation level/density in AML patients was similar to controls (Figure 1B and 1D), and further confirmed by TCGA and GEO data analyzed through online tool DiseaseMeth version 2.0 (Figure 1C). Recently, *ID4* was identified as the direct target of *miR-335* [18]. Moreover, our previous study focused on *ID4* methylation in myeloid malignancies, and revealed that *ID4* expression was downregulated (Figure 1E) but not highly correlated with promoter hypermethylation [19]. Here, we further observed the negative correlation between *miR-335* and *ID4* expression (Figure 1F).

### Aberrant miR-335/ID4 expression affected chemotherapy response in AML

In order to analyze the association of *miR-335*/*ID4* with clinical characteristics of AML, total patients were firstly divided according to *miR-335* expression (the cutoff value 0.048 was based on the ROC curve, the sensitivity and specificity were 55.5% and 73.9%, groups named *miR-335*^high^ and *miR-335*^low^) and *ID4* expression (the cutoff value 0.017 was based on the ROC curve, the sensitivity and specificity were 45.2% and 95.3%, groups named *ID4*^high^ and *ID4*^low^). Finally, we further divided patients into three groups (*miR-335*^low^*ID4*^high^, *miR-335*^low^*ID4*^low^/*miR-335*^high^*ID4*^high^, and *miR-335*^high^*ID4*^low^) to determine the clinical significance. No significant differences were observed between *miR-335*/*ID4* expressions with all the patient's parameters except for complete remission (CR) (Table 1). The rate of CR in patients with *miR-335*^high^*ID4*^low^ was the lowest (34.1%, 14/41), higher in patients with *miR-335*^low^*ID4*^low^/*miR-335*^high^*ID4*^high^ (40%, 26/65), and the highest in patients with *miR-335*^low^*ID4*^high^ (62.5%, 25/40). Also, significant differences were also observed among both non-M3-AML and CN-AML (Table 1). Furthermore, Logistic regression analysis further confirmed that *miR-335*/*ID4* expression independently affected CR in both non-M3-AML and CN-AML after receiving induction therapy (Table 2).

**Table 1:** Comparison of clinical/laboratory features of AML patients with aberrant *miR-335*/*ID4* expression.

**Patient's parameters**	***miR-335*^low^*ID4*^high^** (n=40)**	***miR-335*^high^*ID4*^high^ or *miR-335*^low^*ID4*^low^** (n=65)**	***miR-335*^high^*ID4*^low^ (n=41)**	***P* value**
Sex, male/female	20/20	39/26	26/15	0.443
Age, median (range), years	58.5 (15-93)	55 (17-81)	61 (14-87)	0.286
WBC, median (range), ×10^9^/L	6.7 (0.3-528.0)	12.2 (0.8-201.0)	27.8 (0.9-136.1)	0.253
Hemoglobin, median (range), g/L	76 (42-133)	75 (32-142)	78 (40-126)	0.377
Platelets, median (range),×10^9^/L	41 (5-447)	42 (3-399)	33 (7-234)	0.623
BM blasts, median (range), %	47.5 (1.0-97.5)	42.5 (5.5-94.5)	35.0 (3.0-99.0)	0.893
FAB subtypes				0.399
M0	0 (0%)	1 (2%)	0 (0%)	
M1	1 (3%)	4 (6%)	4 (10%)	
M2	16 (40%)	28 (43%)	15 (36%)	
M3	7 (17%)	13 (20%)	9 (22%)	
M4	7 (17%)	15 (23%)	9 (22%)	
M5	8 (20%)	2 (3%)	4(10%)	
M6	1 (3%)	2 (3%)	0 (0%)	
Karyotypes				0.493
normal	19 (47%)	30 (46%)	20 (49%)	
t(8;21)	4 (10%)	4 (6%)	0 (0%)	
t(15;17)	7 (17%)	13 (20%)	9 (22%)	
+8	1 (3%)	3 (5%)	1 (2%)	
-5/5q-	0 (0%)	2 (3%)	1 (2%)	
-7/7q-	0 (0%)	1 (2%)	0 (0%)	
others	4 (10%)	3 (5%)	6 (16%)	
complex	4 (10%)	8 (11%)	1 (2%)	
No data	1 (3%)	1 (2%)	3 (7%)	
Gene mutations				
*CEBPA* (+/-)	4/32	6/55	5/32	0.938
*NPM1* (+/-)	4/32	8/53	3/34	0.735
*FLT3*-ITD (+/-)	6/30	6/55	5/32	0.570
*c-KIT* (+/-)	2/34	2/59	0/37	0.286
*NRAS*/*KRAS* (+/-)	5/31	5/56	1/36	0.214
*IDH1*/*2* (+/-)	4/32	3/58	2/35	0.511
*DNMT3A* (+/-)	3/33	4/57	3/34	0.920
*U2AF1* (+/-)	1/35	3/58	2/35	1.000
*SRSF2* (+/-)	2/34	3/58	1/36	0.880
CR (+/-), whole-cohort	25/15	26/39	14/27	0.024
CR (+/-), non-M3-AML	18/15	16/36	8/24	0.029
CR (+/-), CN-AML	13/6	11/19	5/15	0.018

WBC: white blood cells; BM: bone marrow; FAB: French-American-British classification; CR: complete remission; non-M3-AML: acute myeloid leukemia without FAB-M3; CN-AML: cytogenetically normal AML.

**Table 2:** Multivariate analyses of variables for overall survival in AML patients.

**Variables**	**Non-M3-AML**				**CN-AML**			
	**CR**		**OS**		**CR**		**OS**	
	**OR (95% CI)**	***P***	**HR (95% CI)**	***P***	**OR (95% CI)**	***P***	**HR (95% CI)**	***P***
*MiR-335/ID4*	2.010 (1.057–3.823)	0.033	1.894 (1.392–2.579)	0.000	3.224 (1.335–7.784)	0.009	2.376 (1.574–3.586)	0.000
Age	4.079 (1.594–10.435)	0.003	1.481 (0.945–2.320)	0.087	3.915 (1.206–12.703)	0.023	1.770 (0.970–3.231)	0.063
WBC	1.469 (0.532–4.054)	0.458	1.415 (0.917–2.183)	0.117	2.056 (0.536–7.885)	0.293	1.551 (0.853–2.820)	0.150
Karyotype	4.214 (1.508–11.774)	0.006	1.595 (1.143–2.227)	0.006	–	–	–	–
*CEBPA* mutations	0.529 (0.132–2.114)	0.367	0.796 (0.405–1.565)	0.509	2.293 (0.365–14.393)	0.376	1.377 (0.559–3.394)	0.487
*NPM1* mutations	1.144 (0.227–5.765)	0.871	0.972 (0.449–2.101)	0.942	1.955 (0.289–13.204)	0.492	1.383 (0.558–3.427)	0.484
*FLT3*–ITD mutations	2.428 (0.541–10.897)	0.247	1.141 (0.567–2.294)	0.712	3.598 (0.610–21.229)	0.157	1.080 (0.380–3.068)	0.885
*C–KIT* mutations	3.130 (0.139–70.308)	0.472	1.523 (0.340–6.824)	0.583	0.785 (0.010–64.537)	0.914	1.181 (0.146–9.894)	0.876
*N/K–RAS* mutations	7.147 (1.137–44.916)	0.036	1.902 (0.917–3.944)	0.084	12.082 (1.001–145.767)	0.050	2.904 (1.061–7.948)	0.038
*IDH1/2* mutations	3.746 (0.349–40.186)	0.275	2.903 (1.289–6.537)	0.010	6.979 (0.534–91.233)	0.139	3.154 (1.248–7.969)	0.015
*DNMT3A* mutations	0.651 (0.104–4.076)	0.646	0.914 (0.408–2.048)	0.827	0.410 (0.050–3.346)	0.405	0.652 (0.262–1.624)	0.358
*U2AF1* mutations	undetermined	0.999	1.815 (0.751–4.386)	0.186	undetermined	0.999	1.480 (0.423–5.179)	0.539
*SRSF2* mutations	undetermined	0.999	1.906 (0.788–4.610)	0.153	undetermined	0.999	1.141 (0.215–6.057)	0.877

CR, complete remission; OS, overall survival; OR, odd ratio; HR, hazard ratio; CI, confidence interval. Variables in multivariate analysis including *miR-335/ID4* expression (Low vs. High), age (≤60 vs. >60 years), WBC (≥30×10^9^ vs. <30×10^9^ /L), karyotype (favorable vs. intermediate vs. poor), and gene mutations (mutant vs. wild-type).

### Dysregulated miR-335/ID4 expression predicted clinical outcome in AML

**Figure 2:**
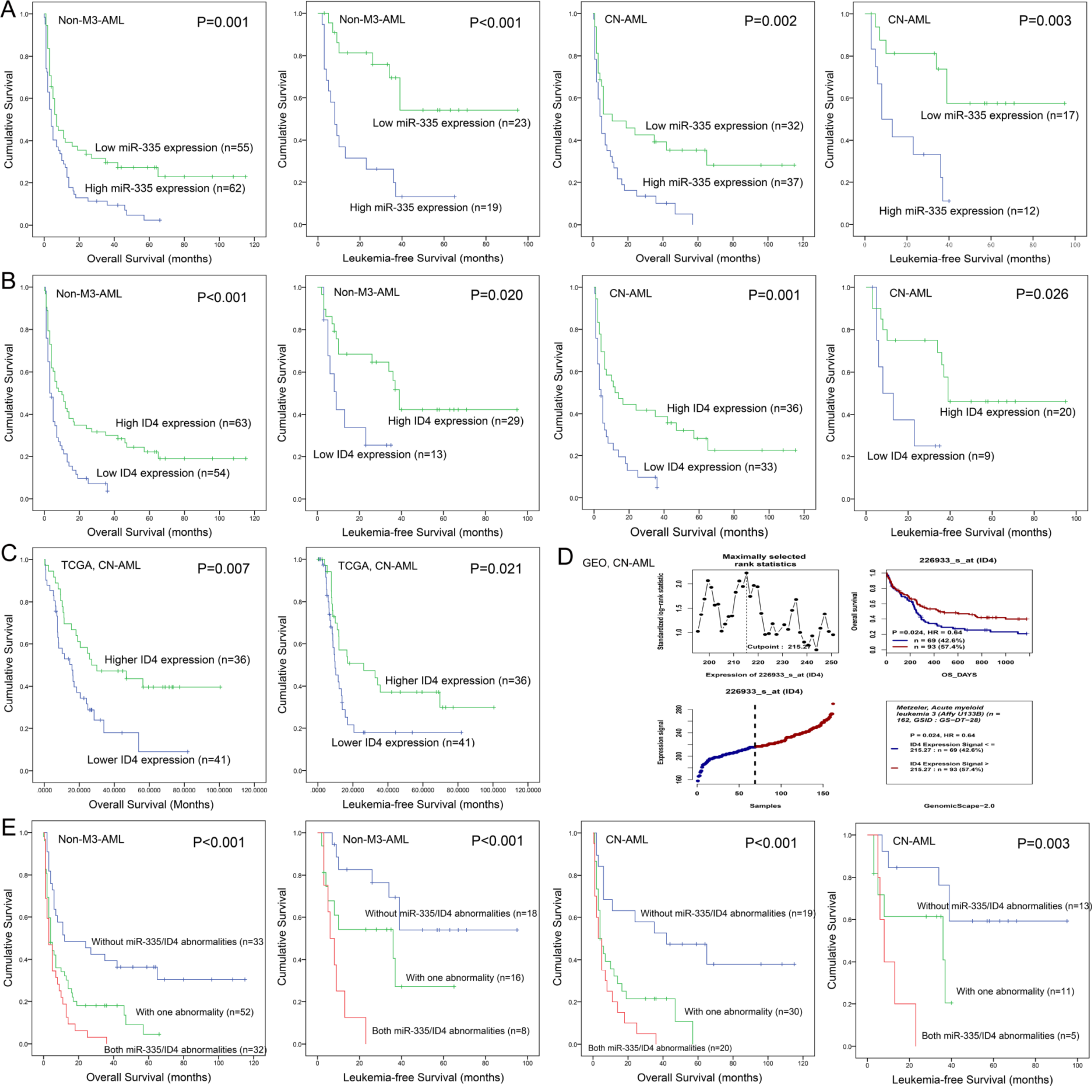
**Aberrant *miR-335/ID4* expression predicted clinical outcome in AML.** (**A**) The prognostic value of *miR-335* expression for OS and LFS in non-M3-AML and CN-AML patients. (**B**) The prognostic value of *ID4* expression for OS and LFS in non-M3-AML and CN-AML patients. (**C**) The prognostic value of *ID4* expression for OS and LFS among CN-AML patients based on TCGA databases. (**D**) The prognostic value of *ID4* expression for OS among CN-AML patients obtained by bioinformatics analysis. The effect of *ID4* expression on prognosis was determined by the Genomicscape (http://genomicscape.com/microarray/survival.php). (**E**) The prognostic value of combined *miR-335/ID4* expression for OS and LFS in non-M3-AML and CN-AML patients.

Due to independent disease entity, AML-M3/acute promyelocytic leukemia (APL) was excluded from the survival analysis. Firstly, we determined the prognostic value of *miR-335* and *ID4* expression in AML, respectively. Patients with *miR-335*^high^ presented significantly shorter LFS and OS than those with *miR-335*^low^ in both non-M3-AML and CN-AML (Figure 2A). Similarly, *ID4*^low^ patients also showed significantly shorter LFS and OS than those with *ID4*^high^ among both non-M3-AML and CN-AML (Figure 2B). Moreover, the prognostic impact of *ID4* expression on OS among CN-AML patients was also confirmed by TCGA data and GEO data analyzed though online tool GenomicScape (Figure 2C and 2D). Secondly, we further evaluated the prognostic significance of *miR-335*/*ID4* expression for LFS and OS based on the three groups (*miR-335*^low^*ID4*^high^, *miR-335*^low^*ID4*^low^/*miR-335*^high^*ID4*^high^, and *miR-335*^high^*ID4*^low^), and the statistic value was enhanced (Figure 2E). Moreover, Cox regression multivariate analysis further confirmed that *miR-335*/*ID4* expression was an independent prognostic biomarker for OS among both non-M3-AML and CN-AML (Table 2).

### MiR-335 exhibited pro-proliferative and anti-apoptotic effects in leukemic cell-lines

**Figure 3:**
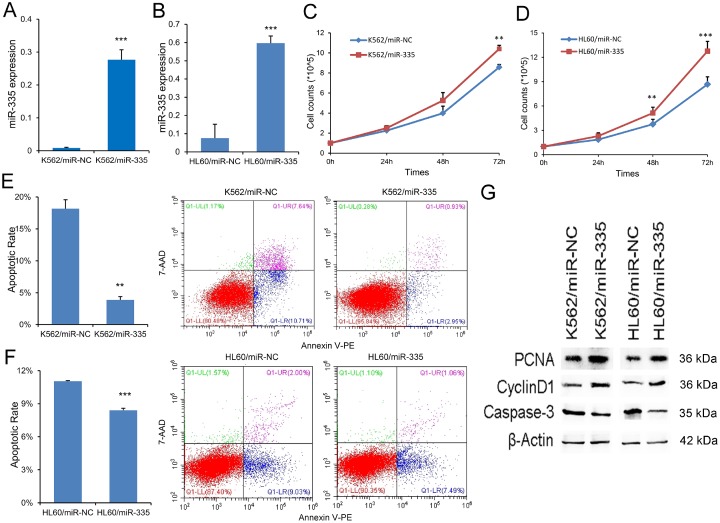
***MiR-335* exhibited pro-proliferative and anti-apoptotic effects in leukemic cell-lines.** (**A–B**) Confirmation of *miR-335* expression after *miR-335* transfection. *MiR-335* expression was significantly upregulated after *miR-335* transfection in both K562 and HL60 cell-lines. (**C–D**) The effect of *miR-335* overexpression on cell proliferation. Overexpression of *miR-335* significantly increased the proliferation ability in K562 and HL60 cell-lines. (**E–F**) The effect of miR-335 overexpression on cell apoptosis. Overexpression of miR-335 significantly decreased the apoptosis ratio in K562 and HL60 cell-lines. (**G**) The expression of proliferation-related proteins (PCNA and Cyclin D1) and apoptosis-related proteins (Caspase-3) affected by *miR-335* overexpression. The expression of proliferation-related proteins (PCNA and Cyclin D1) was increased, whereas the apoptosis-related proteins (Caspase-3) expression was decreased after *miR-335* overexpression in K562 and HL60 cell-lines. *, *P*<0.05; **, *P*<0.01; ***, *P*<0.001.

To investigate the biological role of *miR-335* in AML, gain-of-function experiments were performed in leukemic cell-lines K562 and HL60 since they showed low *miR-335* expression. We transfected *miR-335* plasmid into K562 and HL60 (called K562/*miR-335* and HL60/*miR-335*, controls as K562/miR-NC and HL60/miR-NC), and *miR-335* overexpression was confirmed by RT-qPCR (Figure 3A and 3B). Overexpression of *miR-335* significantly increased the proliferation ability in K562 and HL60 cell-lines (Figure 3C and 3D), together with increased expression of proliferation-related proteins PCNA and Cyclin-D1 (Figure 3G). Moreover, the ratio of apoptosis was significantly reduced in K562 and HL60 cell-lines when overexpressing *miR-335* (Figure 3E and 3F), and confirmed by decreased expression of apoptosis-related Caspase-3 (Figure 3G). In addition, we did not observe the effect of *miR-335* overexpression on cell differentiation based on the expression of CD11b (data not shown).

### ID4 rescued the pro-leukemia effects of miR-335 in leukemic cell-lines

**Figure 4:**
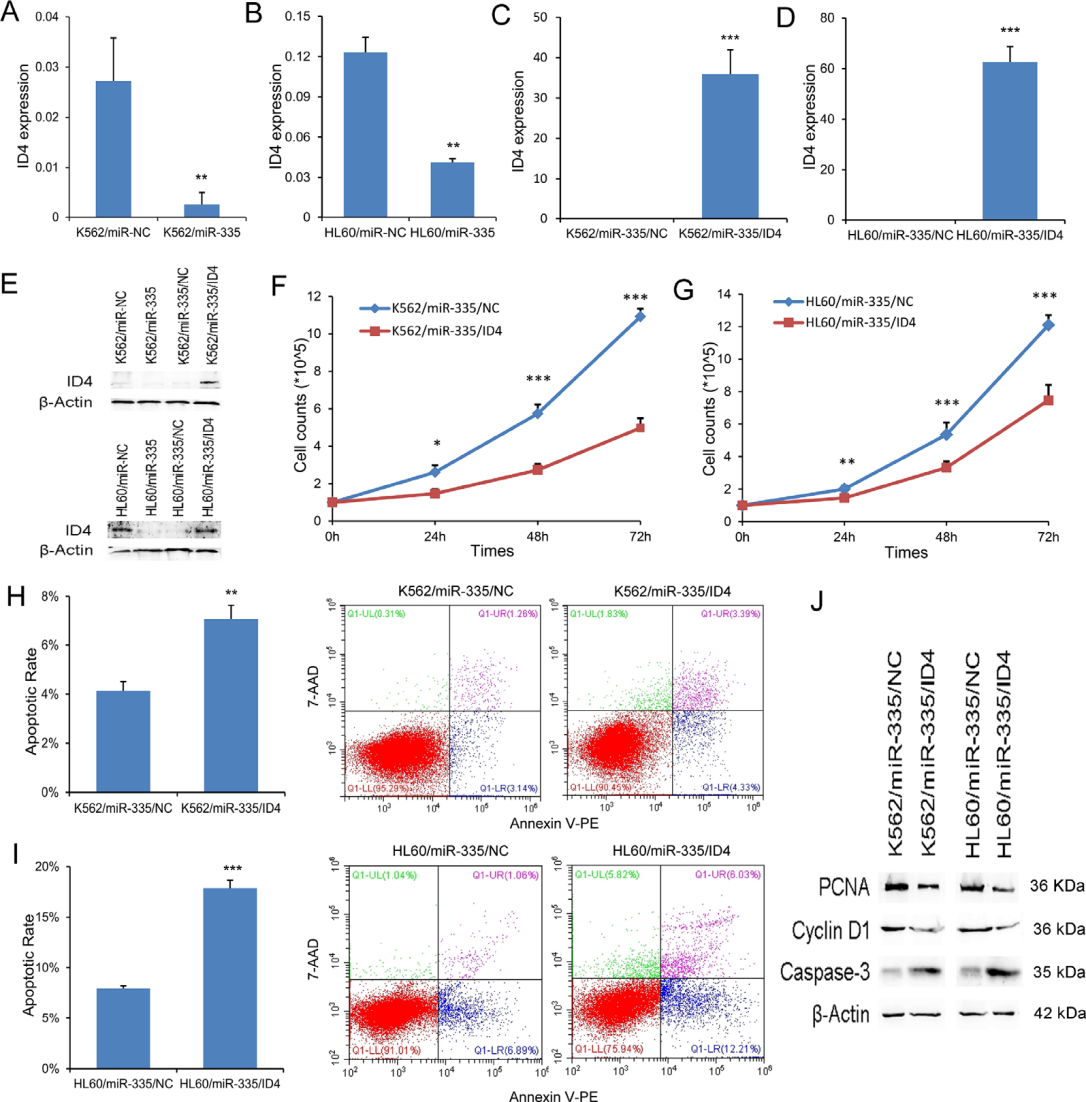
***ID4* rescued the pro-leukemia effects of *miR-335* in leukemic cell-lines.** (**A–B**) *ID4* mRNA expression affected by *miR-335* transfection. *ID4* mRNA expression was significantly reduced after *miR-335* overexpression in both K562 and HL60 cell-lines. (**C–D**) Confirmation of *ID4* mRNA expression after *ID4* restoration. *ID4* mRNA expression was significantly upregulated after *ID4* transfection in both K562/miR-335 and HL60/miR-335 cells. (**E**) ID4 protein expression affected by *miR-335* overexpression and *ID4* restoration. *ID4* protein expression was significantly reduced after *miR-335* overexpression in both K562 and HL60 cell-lines, and was increased after *ID4* restoration. (**F**–**G**) The effect of *ID4* restoration on cell proliferation. Restoration of *ID4* significantly reduced the proliferation ability in K562/miR-335 and HL60/miR-335 cells. (**H**–**I**) The effect of *ID4* restoration on cell apoptosis. Restoration of *ID4* significantly increased the apoptosis ratio in K562/miR-335 and HL60/miR-335 cells. (**J**) The expression of proliferation-related proteins (PCNA and Cyclin D1) and apoptosis-related proteins (Caspase-3) affected by *ID4* restoration. The expression of proliferation-related proteins (PCNA and Cyclin D1) was decreased, whereas the apoptosis-related proteins (Caspase-3) expression was increased after *ID4* restoration in K562/miR-335 and HL60/miR-335 cells. *, *P*<0.05; **, *P*<0.01; ***, *P*<0.001.

Emerging evidence showed that *ID4* was direct target of *miR-335*, acting as a tumor suppressor in leukemia. Herein, we also observed that *ID4* expression at mRNA and protein level was significantly reduced by *miR-335* overexpression in both K562 and HL60 (Figure 4A, 4B, and 4E). In order to determine the role of *ID4* in the process of leukemogenesis caused by *miR-335* overexpression, we restored *ID4* expression in K562/*miR-335* and HL60/*miR-335* cells. *ID4* overexpression was confirmed by RT-qPCR and western blot (Figure 4C–4E). Restoration of *ID4* expression significantly inhibited the proliferation ability in K562/*miR-335* and HL60/*miR-335* cells (Figure 4F and 4G), at the same time, with decreased expression of proliferation-related proteins PCNA and Cyclin-D1 (Figure 4J). Moreover, the apoptosis ratio was significantly upregulated in K562/*miR-335* and HL60/*miR-335* cells when overexpressing *ID4* (Figure 4H and 4I), and confirmed by increased expression of apoptosis-related Caspase-3 (Figure 4G).

### MiR-335/ID4 contributed to leukemogenesis through PI3K/Akt signaling pathway

**Figure 5:**
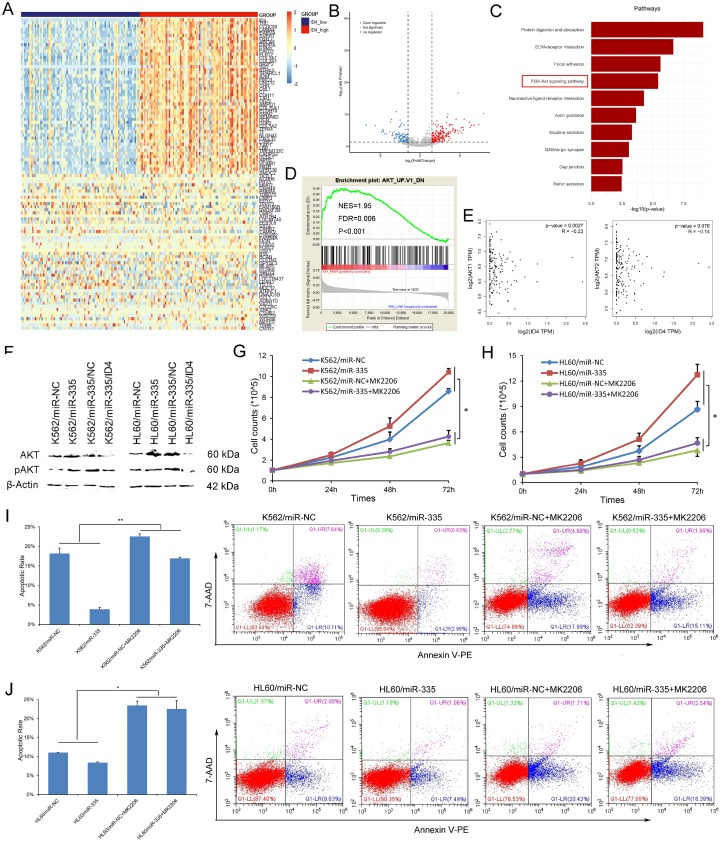
***MiR-335* contributed to leukemogenesis through PI3K/Akt signaling pathway.** (**A**) Expression heatmap of top 50 differentially expressed genes (DEGs) between *ID4*^low^ and *ID4*^high^ AML patients among TCGA datasets (FDR<0.05, *P*<0.05 and |log2 FC|>1.5). (**B**) Volcano plot of DEGs. (**C**) Significantly enriched pathway terms of DEGs in AML. DEGs functional and signaling pathway enrichment was conducted using online website of STRING (http://string-db.org). (**D**) GSEA showed the significantly enriched in genes down-regulated in mouse prostate by transgenic expression of human *AKT1* gene in *ID4*^high^ AML. (**E**) The association between *ID4* expression and *AKT1/AKT2* expression in AML among TCGA datasets. A negative correlation was observed between *ID4* and *AKT1/AKT2* expression. The correlation analysis conducted through online web GEPIA (http://gepia.cancer-pku.cn/detail.php?clicktag=correlation) using Spearman test. (**F**) The expression of Akt and pAkt affected by *miR-335* overexpression and *ID4* restoration. Akt and pAkt protein expression was significantly increased after *miR-335* overexpression in both K562 and HL60 cell-lines, and was decreased after *ID4* restoration. (**G–H**) The effect of *miR-335* on proliferation of K562 and HL60 cell-lines with/without Akt inhibitor MK2206 2HCL. MK2206 2HCL remarkably reversed and impaired the pro-proliferative effect in K562 and HL60 cell-lines. (**I–J**) The effect of miR-335 on apoptosis of K562 and HL60 cell-lines with/without Akt inhibitor MK2206 2HCL. MK2206 2HCL remarkably reversed and impaired the pro-proliferative effect in K562 and HL60 cell-lines. *, *P*<0.05; **, *P*<0.01; ***, *P*<0.001.

To further identify the biological network and potential mechanism involved in the pro-leukemia effects of *miR-335*/*ID4*, we first analyzed the differentially expressed genes (DEGs) between *ID4*^low^ and *ID4*^high^ groups of AML patients among TCGA datasets. A total of 998 DEGs (892 positively correlated and 106 negatively correlated, FDR<0.05, *P*<0.05 and |log2 FC|>1.5) were identified (Figure 5A and 5B). To gain further insight into the function of identified DEGs, functional and pathway enrichment analysis was performed. We found that the DEGs mainly involved in PI3K/Akt signaling pathway (Figure 5C). Moreover, GSEA showed the significantly enriched in genes down-regulated in mouse prostate by transgenic expression of human *AKT1* gene in *ID4*^high^ AML (Figure 5D). In addition, we also observed that the negative association between *ID4* and *AKT1/AKT2* expression in AML patients among TCGA datasets (Figure 5E, Spearman test).

Next, we confirmed that *miR-335* overexpression significantly upregulated the expression of both Akt and pAkt, which was reversed by *ID4* overexpression in both K562 and HL60 leukemic cell-lines (Figure 5F). To further validate whether PI3K/Akt signaling was involved in the pro-leukemia effects of *miR-335* overexpression on K562 and HL60 cell-lines, MK-2206 2HCL which is an inhibitor of Akt was further used to block PI3K/Akt signaling pathway. We found that MK-2206 2HCL remarkably reversed and impaired the pro-proliferative and anti-apoptotic effects in K562 and HL60 cell-lines (Figure 5G–5J). All the results suggested that *miR-335/ID4* contributed to leukemogenesis through activating PI3K/Akt signaling pathway.

## DISCUSSION

*MiR-335*, which is transcribed from the chromosome 7q32.2, has been observed to aberrantly express in diverse cancers including hematological malignances and to play a crucial role in cancer initiation and progression [12]. *MiR-335* was identified to be involved in tumor proliferation, apoptosis, migration, invasion and metastasis by targeting multiple genes and some and some cell signaling pathways in vitro or/and in vivo [12]. the present study, we found that *miR-335* was overexpressed in AML patients, and increased expression of *miR-335* expression was negatively associated with decreased expression of *ID4*, which acted as tumor suppressor in AML [19]. Further functional studies demonstrated that *miR-335* exhibited pro-proliferative and anti-apoptotic effects by targeting *ID4* in AML. Two recent studies also showed that overexpression of *miR-335* was a frequent event in both adult and pediatric AML [13, 14]. Similarly, *miR-335* was also a promoter of neurologic tumors and multiple myeloma [20–22]. In contrast, *miR-335* was frequently downregulated and functioned mainly as a suppressor in a majority of the solid tumors, such as hepatocellular cancer, prostate cancer, clear cell renal cell carcinoma, colorectal cancer, breast cancer, cervical cancer, ovarian cancer, and lung cancer [12]. The conflicting results showed that *miR-335* played either as tumor suppressor genes or oncogenes mainly depending on various cancer types.

With progress in genomics technology has tremendously increased our knowledge of the molecular heterogeneity of AML, and has these insights been translated into improved disease classification, clinical care, and novel therapeutic approaches [23, 24]. Herein, we showed that combined *miR-335* and *ID4* expression was associated chemotherapy response in AML. Several studies showed the direct role of *miR-335* in regulating drug resistance. Martin et al. reported that *miR-335* played an oncogenic role in promoting agonistic estrogen signaling in a cancerous setting, and resulted in the enhanced resistance of MCF-7 cells to the growth inhibitory effects of tamoxifen [25]. Moreover, *miR-335* was identified to regulate the chemo-radioresistance of small cell lung cancer cells by targeting PARP-1 [26]. Kim et al. also revealed that *miR-335* conferred sensitivity to anti-cancer drugs by increasing the expression of HDAC3 [27]. As a result, it was not surprising that *miR-335*/*ID4* expression correlated with clinical outcome. Similarly, the effect of *miR-335* overexpression on both CR and OS in AML was also confirmed by the two previously studies regarding *miR-335* expression in AML [13, 14]. All the results suggested *miR-335*/*ID4* expression were potential prognostic biomarkers in AML.

The PI3K/Akt signaling pathway is crucial to widely divergent physiological processes including cell proliferation, cell cycle progression, cell apoptosis, cell survival, differentiation, angiogenesis and drug resistance [28]. Approximately 60% of AML patients showed Akt phosphorylated on Thr308 and/or Ser473, indicating the activation of PI3K/Akt signaling is a frequent event in AML [29]. In addition, both LFS and OS were significantly shorter in AML cases where pathway up-regulation was documented [30]. Activation of PI3K/Akt signaling in AML may be resulted from several factors, including mutations of FLT3, c-Kit, and N/K-Ras mutations, PI3K p110β/δ overexpression [31]. Herein, although we did not observe the associations of aberrant *miR-335/ID4* expression with these gene mutations in AML, evidence shwed that *miR-335*/*ID4* expression was associated Akt/pAkt expression in leukemic cell-lines and AML patients, and inhibition of PI3K/Akt signaling reversed and impaired the pro-proliferative and anti-apoptotic effects mediated by *miR-335* overexpression in K562 and HL60 cell-lines. All the results provided new insights into the underlying mechanism of PI3K/Akt activation in AML, and gave potentially novel targets for drug development to treat AML patients in the future.

Taken together, our findings revealed that aberrant *miR-335*/*ID4* expression was a frequent event, and independently affected clinical outcome in AML. Moreover, *miR-335*/*ID4* dysregulation facilitated leukemogenesis through the activation of PI3K/Akt signaling pathway.

## MATERIALS AND METHODS

### Patients and treatment

The present study was approved by Institutional Ethics Committee of the Affiliated People’s Hospital of Jiangsu University. After written informed consents were obtained from all participants, a total of 146 bone marrow (BM) samples collected from AML patient and 46 BM collected from healthy donors were included in this study. The diagnosis and classification of AML patients were established according to the French-American-British (FAB) classification and the revised World Health Organization (WHO) criteria [32, 33]. The characteristics of AML patients were summarized in Table 1. Treatment regimens for AML patients were induction chemotherapy and subsequent consolidation chemotherapy as reported [34, 35].

### Cytogenetic analysis and BMMNCs samples preparation

Karyotypes were analyzed at the newly diagnosis time by conventional R-banding method according to the previous literature [36]. BM mononuclear cells (BMMNCs) were separated by density-gradient centrifugation using Lymphocyte Separation Medium (Absin, Shanghai, China), and used for RNA and DNA extraction once BMMNCs were isolated.

### Cell line and cell culture

Human leukemic cell lines K562 and HL60 were cultured in RPMI 1640 medium (BOSTER, Wuhan, China) containing 10% fetal calf serum (ExCell Bio, Shanghai, China) and grown at 37°C in 5% CO_2_ humidified atmosphere.

### RNA isolation and reverse transcription

Total RNA isolation was conducted as reported previously [37]. Reverse transcription obtained cDNA used for miRNAs was performed using MiScript Reverse Transcription Kit (Qiagen, Duesseldorf, Germany), whereas cDNA used for mRNAs was performed using random primers as reported [19, 38]. cDNA was stored at -80°C until used.

### RT-qPCR

The expression of *miR-335* was detected by real-time quantitative PCR (RT-qPCR) using miScript SYBR green PCR kit (Qiagen, Duesseldorf, Germany). *U6* detected by the same reagent was used to calculate the abundance of *miR-335* level. The primers for *miR-335* and *U6* were listed in Supplementary Table 1. *ID4* expression was examined by RT-qPCR using AceQ qPCR SYBR Green Master Mix (Vazyme Biotech Co., Piscataway, NJ, USA). The housekeeping gene *ABL1* detected by 2×SYBR Green PCR Mix (Multisciences, Hangzhou, China) was used to calculate the abundance of *ID4* transcript. The primers for *ID4* and *ABL1* were shown in Supplementary Table 1. Relative *miR-335* and *ID4* expression was calculated using 2^-ΔΔCT^ method.

### DNA isolation, bisulfite modification, and RQ-MSP

Genomic DNA isolation and modification were performed as reported previously [19], and modified DNA were stored at -80°C until used. The level of *miR-335* methylation was detected by real-time quantitative methylation-specific PCR (RQ-MSP) using AceQ qPCR SYBR Green Master Mix (Vazyme Biotech Co., Piscataway, NJ, USA) with primers showed in Supplementary Table 1. *ALU* detected by 2×SYBR Green PCR Mix (Multisciences, Hangzhou, China) with primers (Supplementary Table 1) was used to calculate the abundance of *miR-335* methylation level. Relative *miR-335* methylation level was calculated using 2^-ΔΔCT^ method.

### BSP

TaKaRa Taq^TM^ Hot Start Version kit (Tokyo, Japan) was used for bisulfite sequencing PCR (BSP) reaction with primers showed in Supplementary Table 1. BSP products cloning sequencing was performed as described [19]. Eight independent clones from each specimen were sequenced (BGI Tech Solutions Co., Shanghai, China).

### Gene mutation detection

Gene mutations including *CEBPA*, *NPM1*, *FLT3*-ITD, *C-KIT*, *NRAS/KRAS*, *IDH1/2*, *DNMT3A*, *U2AF1*, and *SRSF2* were detected by high-resolution melting analysis (HRMA) and/or direct DNA sequencing as reported [39–44]*.*

### Plasmid construction and transfection

Human mature *miR-335* sequences were cloned in pGCMV/EGFP/miR/Blasticidin expression vector, whereas human full-length *ID4* CDS sequences were cloned in PEX-2 expression vector, and all of them were purchased from GenePharma (Shanghai, China). Cell transfection was peformed using HiPerFect Transfection Reagent (Qiagen, Duesseldorf, Germany). *MiR-335*/*ID4* stably expressed cells were selected by Blasticidin and G418 (InvivoGen, San Diego, CA, USA), respectively, and flow sorting (BD FACSAriall, San Jose, CA, USA).

### Treatment with Akt inhibitor MK-2206 2HCL

The Akt inhibitor MK-2206 2HCL (Selleck Chemicals, Houston, TX, USA) was used to block PI3K/Akt signaling in leukemic cell-lines at a final concentrations of 5 μM.

### Western blot

Western blotting was performed as described previously [45]. The antibodies were mouse anti-β-actin (BOSTER, Wuhan, China), rabbit anti-*ID4* (Abcam, Cambridge, MA, USA), rabbit anti-PCNA (Cell Signaling Technology, Danvers, MA, USA), rabbit anti-Cyclin D1 (Cell Signaling Technology, Danvers, MA, USA), rabbit anti-Caspase-3 (Cell Signaling Technology, Danvers, MA, USA), rabbit anti-Akt/pAkt (Beijing Solarbio Science & Technology Co., Ltd., Beijing, China), and anti-mouse/anti-rabbit secondary antibodies (Fcmacs, Nanjing, China).

### Cell proliferation assays

Cells (1×10^5^ cells/ml) were seeded onto a 6-well plate in RPMI 1640 medium containing 10% fetal calf serum. After culturing for 0, 1, 2, and 3 days, cells were counted in counting board for three times.

### Cell apoptosis analysis

Cells (2×10^5^ cells/ml) were seeded onto a 6-well plate in RPMI 1640 medium containing 1% fetal calf serum for 2 days. Annexin V-PE/7-AAD apoptosis detection kits (BD Pharmingen, San Diego, CA, USA) were used to analyze the apoptosis rate according to the manufacturer’s protocols, and then analyzed via flow cytometry (Beckman Coulter, Miami, FL, USA). Each experiment was repeated three times.

### Immunophenotypic analysis by flow cytometry

Flow cytometric analysis was performed to determine surface marker expression. Cells at logarithmic growth phase (5×10^5^ cells/ml) were stained with antibodies in phosphatebuffered saline (PBS) at 4°C for 30 min, and then washed and resuspended in 300 μL FACS buffer. The antibody was anti-human/mouse CD11b PE (Biogems, Rocky Hill, NJ, USA).

### TCGA and GEO datasets

A cohort of 200 AML patients (NEJM 2013) from The Cancer Genome Atlas (TCGA) [23] was downloaded via cBioPortal (http://www.cbioportal.org) [46, 47]. A cohort of 162 cytogenetically normal AML (CN-AML) patients (GSE12417) from Gene Expression Omnibus (GEO) data was also used (https://www.ncbi.nlm.nih.gov/geo/).

### Bioinformatics analyses

The human disease methylation database DiseaseMeth version 2.0 (http://bio-bigdata.hrbmu.edu.cn/diseasemeth/) was used for differential methylation analysis for *miR-335/MEST* (*miR-335* host gene). The prognostic impact of *ID4* expression in CN-AML patients for GSE12417 analyzed through the online web Genomicscape (http://genomicscape.com/microarray/survival.php) [48, 49]. The correlation between *ID4* and *AKT1/AKT2* expression was analyzed through online web GEPIA (http://gepia.cancer-pku.cn/detail.php?clicktag=correlation) [50]. Gene Set Enrichment Analysis (GSEA) was performed using GSEA v3.0 software (http://www.broad.mit.edu/gsea). Functional and signaling pathway enrichment was conducted using online website of STRING (http://string-db.org).

### Statistical analyses

SPSS 20.0 software package (IBM, Armonk, NY, USA) was applied to statistical analyses. Student's T test/Mann-Whitney’s U test was performed to compare the differences of continuous variables. The difference of categorical variables was analyzed using Pearson Chi-square analysis/Fisher exact test. Spearman correlation test was conducted to evaluate the correlation between continuous variables. The receiver operating characteristic (ROC) curve and area under the ROC curve (AUC) were carried out to assess the discriminative capacity of *miR-335/ID4* expression between patients and controls. Kaplan-Meier and Cox regression analysis (univariate and multivariate analysis) were used to analyze the impact of *miR-335*/*ID4* expression on survival. Statistical significance was set at *P*<0.05 and all tests were two sided.

## Supplementary Material

Supplementary Table
