# Supplementary material for: MicroRNA-335/ID4 dysregulation predicts clinical outcome and facilitates leukemogenesis by activating PI3K/Akt signaling pathway in acute myeloid leukemia
Source: Aging (Albany NY). 2019 May 30;11(10):3376–91. doi: 10.18632/aging.101991 (PMC6555456; doi:10.18632/aging.101991)
Supplement: Supplementary Table [file aging-11-101991-s001.pdf]

SUPPLEMENTARY TABLE

Supplementary Table 1. Primers used for RQ-PCR, RQ-MSP and BSP.

	**Primer sequences**
RQ-PCR	
MiR-335-Forward	5′-TTATAAAGCAATGAGACTGATT-3′
MiR-335-Reverse	Manufacturer-provided miScript universal primer
U6-Forward	5′-GTGCTCGCTTCGGCAGCACATATAC-3′
U6-Reverse	5′-AAAATATGGAACGCTTCACGAATTTG-3′
ID4-Forward	5′-CATCCCGCCCAACAAGAAAGTCA-3′
ID4-Reverse	5′-GCCGGGTCGGTGTTGAGCGCAGT-3′
ABL-Forward	5′-TCCTCCAGCTGTTATCTGGAAGA-3′
ABL-Reverse	5′-TCCAACGAGCGGCTTCAC-3′
RQ-MSP	
MiR-335-Methylation-Forward	5′-TCGTGTTTTTGGTGGTTATC-3′
MiR-335-Methylation-Reverse	5′-TAACCGCTCGAACTAAAAAAC-3′
ALU-Forward	5′-TTAGGTATAGTGGTTTATATTTGTAATTTTAGTA-3′
ALU-Reverse	5′-ATTAACTAAACTAATCTTAAACTCCTAACCTCA-3′
BSP	
MiR-335-Bisulfited-Forward	5′-GGGGTTTTGTTTTTTTAATTGT-3′
MiR-335-Bisulfited-Reverse	5′-AAATACCCCAACTCTTTCCTTA-3′

RQ-PCR: real-time quantitative PCR; RQ-MSP: real-time quantitative methylation-specific PCR; BSP: bisulfite sequencing PCR.
